# Functional Gene Diversity of Selected Indigenous Hydrocarbon-Degrading Bacteria in Aged Crude Oil

**DOI:** 10.1155/2020/2141209

**Published:** 2020-07-30

**Authors:** Chioma Bertha Ehis-Eriakha, Chioma Blaise Chikere, Onyewuchi Akaranta

**Affiliations:** ^1^Department of Microbiology, Edo University Iyamho, Uzairue, Edo State, Nigeria; ^2^Department of Microbiology, University of Port Harcourt, Port Harcourt, Rivers State, Nigeria; ^3^Department of Pure and Industrial Chemistry, University of Port Harcourt, Port Harcourt, Rivers State, Nigeria

## Abstract

Crude oil pollution has consistently deteriorated all environmental compartments through the cycle of activities of the oil and gas industries. However, there is a growing need to identify microbes with catabolic potentials to degrade these pollutants. This research was conducted to identify bacteria with functional degradative genes. A crude oil-polluted soil sample was obtained from an aged spill site at Imo River, Ebubu, Komkom community, Nigeria. Bacteria isolates were obtained and screened for hydrocarbon degradation potential by turbidometry assay. Plasmid and chromosomal DNA of the potential degraders were further screened for the presence of selected catabolic genes (C230, Alma, Alkb, nahAC, and PAHRHD_(GP)_) and identified by molecular typing. Sixteen (16) out of the fifty (50) isolates obtained showed biodegradation activity in a liquid broth medium at varying levels. *Bacillus cereus* showed highest potential for this assay with an optical density of 2.450 @ 600 nm wavelength. Diverse catabolic genes resident in plasmids and chromosomes of the isolates and, in some cases, both plasmid and chromosomes of the same organism were observed. The C230 gene was resident in >50% of the microbial population tested, while other genes occurred in lower proportions with the least observed in nahAC and PAHRHD. These organisms can serve as potential bioremediation agents.

## 1. Introduction

Petroleum hydrocarbons have been identified as a significant group of environmental pollutants in technologically advanced countries. Moreover, the supply of petroleum forms the main sources of energy globally, and it constitutes the major form of environmental pollution during the processes of exploration, mining, purifying, conveying, and marketing petroleum products [1, 2]. The high alarming rate of petroleum hydrocarbons build-up in the environment has led to menacing human and ecosystem safety such as rescinding soil structures, a detrimental effect on groundwater quality, and deterring plant growth [3].

Crude oil/petroleum hydrocarbons presents diverse physical properties as a result of their complex mixtures of several individual compounds classified based on polarity as saturates, aromatics, resins, and asphaltenes [4–6]. The different crude oil components have varying susceptibility to microbial mineralization based on the structural arrangement. Alkanes are the most susceptible; following this are light aromatics (MAHs), cycloalkanes, heavy aromatics (PAH), resins, and asphaltenes. Generally, alkanes make up about 50% of crude oil although the source of the oil may alter the concentration [7]. It has been discovered that PAHs are one of the major factors causing the rapid development of cancer as a result of constant exposure to a mixture of highly concentrated PAHs. This has consequently led to the destruction of genetic materials and later stimulates the growth of cancers [8]. Moreover, some PAHs possess numerous health hazards attributes such as genotoxic, carcinogenic, and cytotoxic, especially to aquatic organisms which may be passed onto humans via consumption of sea foods [9].

The high rate of exploration and fabrication activities together with inappropriate waste disposal practices has been recognized as the major source of terrestrial and aquatic pollution. Therefore, it has become an imperative search for a sustainable remediation technique that could help in ecorestoration of these polluted environments, especially total petroleum hydrocarbon and polycyclic aromatic hydrocarbons. The remediation of these polluted environments has become the major research focus in the field of science, engineering, and environmental science [10, 11].

Bioremediation has been discovered as a type of biodegradation that utilizes beneficial microorganisms for detoxification and the complete removal of inorganic and organic-inorganic xenobiotic compounds from heavily polluted soil. The process of microbial bioremediation involves the utilization of microbial enzymes for biodegradation of these harmful substances from the contaminated soil into innocuous substances [12]. The utilization of hydrocarbon-degrading bacteria which are domicile in these hydrocarbon-polluted soil might be a sustainable, cost-effective, eco-friendly biotechnological tool for the ecorestoration of these oil-polluted soils [13]. This process is essential for adequate reduction of accidental hydrocarbon discharges and for effective protection of these limited resources available in several countries [4]. Myriad of research studies have been conducted to justify the recalcitrance of PAHs and other persistent components of crude oil in the environment. Based on documented research findings, diverse groups of the microbial population present in different environmental compartments have shown potentials to degrade low and high-molecular weight (naphthalene, acenaphthene, anthracene, fluoranthene, pyrene, chrysene) hydrocarbons as the energy source using different mechanisms mediated by enzymes (catabolic genes). Prior to the initiation of bioremediation in a polluted site, it is imperative to determine the presence of active microbes with requisite catabolic genes to effectively drive the remediation process. The presence of these genes, type, and location on specific degradative microbes will provide a guide to how the bioremediation process can be enhanced.

Due to high diversity and complexity of hydrocarbons, degradation of each component is activated by a specific enzyme, for example, the nahAC gene codes for the breakdown of naphthalene and alkB codes for alkanes, while phnAC codes for phenanthrene degradation. Numerous reviews have documented the physiological and enzymatic mechanisms microbes use in the mineralization and degradation of petroleum hydrocarbons [14–16] and observed that different enzymes code for the degradation pathways. An aerobic degradation pathway for petroleum hydrocarbon requires oxygenase enzyme which incorporates oxygen atoms into the hydrocarbons. One or two oxygen atoms can be introduced into the substrate referred to as monooxygenases or dioxygenases, respectively. Oxygen, as an electron acceptor, facilitates the process of aerobic degradation than anaerobic degradation which uses alternate electron acceptors such as nitrate [17–20]. The pathway for aliphatic degradation first produces alcohol that is oxidised through dioxygenases to carboxylic acids which subsequently goes through *β*-oxidation to produce CO_2_ and water. Alternatively, aromatic hydrocarbon degradation occurs through the hydroxylation of a ring mediated by mono- or dioxygenase enzymes to produce diol. Afterwards, the ring is cleaved and further degraded to form catechol or a structurally related compound. Once formed, catechol can be degraded resulting in compounds that can enter into the citric acid (TCA) cycle. The end point of this process is also CO_2_ and water [18–20].

The application of phylogenetic analyses in the study of whole bacteria diversity using 16S rRNA genes is very essential although this technique may not reveal the specific key players involved in petroleum hydrocarbon degradation, as well as highlights the relationship between the microbial community structure and function. However, an alternative approach to this is the integration of I6S rRNA gene information with the results of functional catabolic gene diversity responsible for the mineralization process [21]. This approach reduces the challenges associated with studying the hydrocarbon-degrading microbial community structure, especially highlighting the link between the microbial community and its functions.

In view of those mentioned above, this study scrutinized the functions of various bacterial communities responsible for the degradation of petroleum hydrocarbons and their specific degradative functions. In addition, we isolated, identified, and characterized the indigenous hydrocarbon utilizing bacteria and their community dynamics which are responsible for the biodegradation of petroleum hydrocarbons using culture-dependent and molecular techniques.

## 2. Materials and Methods

### 2.1. Sampling and Soil Characterization

**Figure 1:**
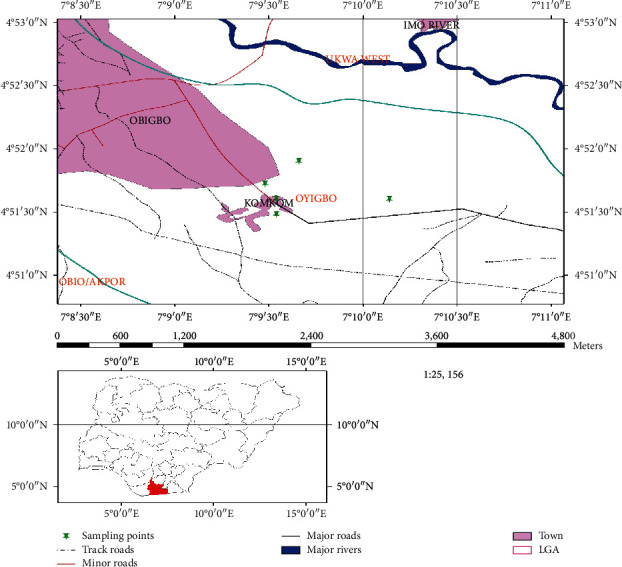
Komkom Community, Oyibo LGA of Rivers State showing the different sampling points.

Crude oil-polluted soil was collected from an aged spill site at Imo River, Ebubu, Komkom community, Rivers State, Nigeria (Figure 1), using a soil auger machine at different depths (0.5 m, 1 m, 1.5 m, and 2 m) from different points. Soil samples were bulked together for homogeneity and transferred to the laboratory for analysis within 6h of collection. Preliminary characteristics of the soil were evaluated using the standard method described by the American Public Health Association (APHA) 20^th^ Edition 2008, American Society for Testing and Materials (ASTM) 2^nd^ Edition 2007, and Environmental Protection Agency (EPA, 2003).

### 2.2. Determination of Total Petroleum Hydrocarbons (TPH) and Polycyclic Aromatic Hydrocarbons (PAH)

Residual TPH and PAH were extracted from the samples and quantified using a gas chromatograph-flame ionization detector (GC-FID). Residual TPHs were extracted with 20 ml of n-pentane from 10 g of each soil sample. The samples were shaken for 15 min by using the ultrasonic apparatus and allowed to settle for 60 min at room temperature. Organic extract (10 ml) was transferred into a vial and analyzed by GC-FID. GC-FID analysis of TPH and PAH was performed on a Hewlett Packard 5890 Series II-Plus gas chromatograph equipped with an HP 7673 autosampler and FID detector coupled with a 30′0.32 mm DB-5 (5% phenyl, 95% methyl-polysiloxane) fused silica capillary column. The oven temperature was programmed from 40°C (3 min.) to 300°C at 15°C/min. Samples were injected in the splitless mode, with the relay open at 20S. Injector and detector temperatures were 250 and 320°C, respectively. Helium was used as the carrier gas at a linear velocity of 38 cm sec^−1^ (15 psig). Data handling was performed with Agilent Chemstation chromatography software (version 10). For quantification purposes, the peak area for TPH was determined using forced line integration with Agilent Chemstation software between *n*-hexane (*n*-C6) through *n*-pentatriacontane (*n*-C35) or until the last peak eluted in the chromatographic profile. For individual PAH, the area of each peak was calculated using the baseline-baseline mode and external response factor quantization [22].

### 2.3. Enumeration of Total Culturable Heterotrophic and Hydrocarbon-Utilizing Bacteria (THB and THUB)

For THB counts, 1g of soil (wet weight) was homogenized in normal saline (0.85%) with a vortexing machine. Ten-fold decimal dilutions of the suspensions were plated out on Plate Count Agar and incubated at 30°C for 24 h. For THUB counts, culture enrichment was performed by adding 1g of soil into 100 ml of Bushnell Haas Mineral Salts (BHMS) amended with 0.5% crude oil (v/v) as the carbon source. The medium was incubated for 5 days at 37°C, 130 rpm for 5 days in a shaker incubator. Thereafter, serial dilution was performed, and the inoculum was plated out on Bushnell Haas agar amended with crude oil for 5 days [21]. Distinct colonies were enumerated and subcultured into nutrient agar plates for further analysis.

### 2.4. Determination of Bacterial Hydrocarbon Degradation Potential by Turbidometry

Turbidometry screening was conducted to determine the degradation potentials of the individual putative hydrocarbon-utilizing bacterium. Representative bacterial isolates were screened for oil degrading capability under aerobic conditions by inoculating a calibrated loop full of 18-hr old culture of each isolate into Bushnell Haas Mineral Salt Broth containing 1% Bonny light crude oil in a shaker incubator (150 rpm) at 30°C for 21 days. Optical density readings, which are a function of biomass increase, were taken at days 0, 3, 6, 11, 16, and 21, respectively, using a calibrated spectrophotometer at 600 nm wavelength against water as blank. Biodegradation potential of each bacterium was scored by turbidity of the media and emulsification of oil in a mineral broth medium and by visual observation of breakdown of the crude oil layer [23].

### 2.5. DNA Extraction and PCR

Genomic DNA and the plasmid used during this study were obtained using the Zymo-Spin Research DNA extraction kit™ (Inqaba Biotech., Pretoria, South Africa). The 16S rRNA (ribonucleic acid) gene amplification and Sanger sequencing techniques were employed to identify the bacterial isolates and characterize the total bacterial community structure in the various microcosms at intervals. PCR was conducted with an Eppendorf thermal cycler (vapo.protect) machine on all DNA extracted samples using universal primer set 27F and 1492R which amplify approximately 1500 bp of the 16S rRNA gene sequence targeting variable regions VI and V3 of the 16S rRNA gene. The 20 *μ*l PCR mixture contained 2 *μ*l volume of the extracted DNA, 0.16 *μ*l each of 20 pmol of both forward 27F 3′GAGTTTGATCCTGGCTCAG 5′ and reverse primers 1492R 5′CTCAAACTAGGCCAGTC 3′, 10 *μ*l of Green Master mix (Taq polymerase, PCR buffer, MgCl_2_, and dNTPs), and 7.68 *μ*l of nuclease-free water. A reaction without template DNA was included as negative control. The PCR program was as follows: a denaturing step at 94°C for 5 minutes followed by 30 cycles of 94°C for 1 minute, annealing temperature for 1 minute at 55°C, extension at 72°C for 1 minute, and final extension at 72°C for 10 minutes and was held at 4°C. Amplified DNA was examined by electrophoresis in 1.5% agarose gel with 2 *μ*l aliquots of PCR products in 1x Tris-Acetate-EDTA buffer and visualized on a UV-trans illuminator.

### 2.6. Determination of Functional Genes in Plasmid and Chromosomal DNA

Five functional degradative genes targeting alk B (short and middle chain alkanes, C_8_–C_16_), Alma (long chain alkane, C_16_–C_36_), catechol 2, 3 dioxygenase (aromatic hydrocarbons), PAH-RHD_(GP)_ (aromatic hydrocarbons), and nah AC (naphthalene degrading gene) were used to detect these genes in the putative hydrocarbon utilizing bacteria to determine their catabolic potentials and capabilities. Also, all isolates were analyzed for plasmid and chromosomal DNA to determine the location of the genes. The process involved PCR with the specific genes targeting specific sites on the bacterial genomic and plasmid DNA molecules responsible for the breakdown of different crude oil constituents. PCR programme for all functional gene amplification is shown in Supplementary Tables 1–4.

### 2.7. Sequencing

Amplified PCR products were purified and sequenced with universal primer set 27F and 1492R using an ABI 3130 XL genetic analyzer incorporating the ABI Big Dye Terminator Cycle sequencing kit version 3.1. Sequencing was performed by Inqaba Biotech, Pretoria, South Africa. The comparison of the nucleotide sequences of the unique fragment with the sequences available in the GenBank database was carried out using the NCBI BLAST program (http//www.ncbi.nlm.nih.gov/blast).

## 3. Results

### 3.1. Baseline Properties of the Samples

**Figure 2:**
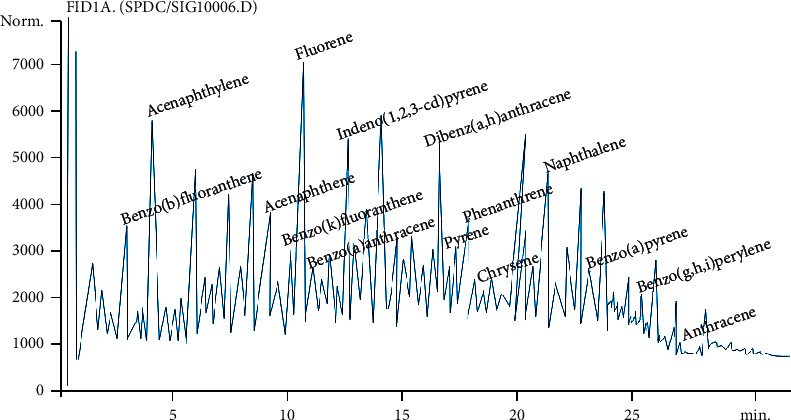
Baseline chromatogram of the polycyclic aromatic hydrocarbons profile of the soil sample.

**Figure 3:**
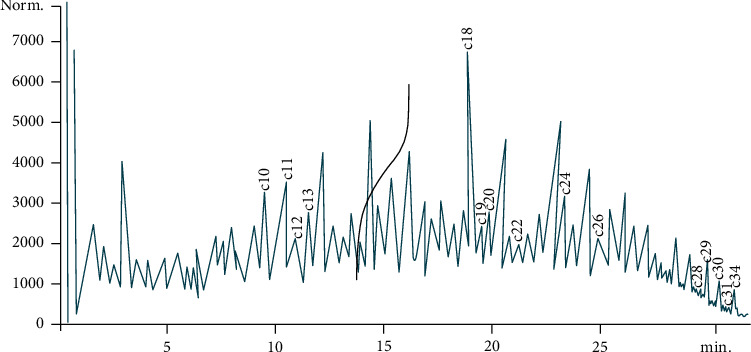
Baseline chromatogram of the total petroleum hydrocarbons profile of the soil sample.

The representative samples of the crude oil-polluted soil were analyzed using physicochemical, gas chromatographic, and microbiological analysis to ascertain the extent of contamination, and GC-FID revealed detectable concentrations of TPH and PAH values as 8695.7723 mg/kg and 989.1188 mg/kg, respectively, as shown in the chromatograms (Figures 2 and 3).

The TPH components in the soil ranged from C10 to C34 with varying concentrations of carbon components (Table 1), while PAH components were benzo(b)fluoranthene (56.94), acenaphthylene (45.09), benzo(k)fluoranthene (78.93), fluorine (197.62), acenaphthene (58.77), benzo(a)anthracene (115.84), indeno (1,2,3 – d)pyrene (67.56), dibenz(a,h)anthracene (26.32), pyrene (100.63), phenanthrene (78.85), chrysene (20.32), naphthalene (17.76), benzo(a)pyrene (45.07), benzo(g, h,i)perylene (25.17), and anthracene (13.00) (Table 2). Other parameters analyzed include pH (5.97), E.C. (60 *μ*s/cm), moisture content (2.4%), nitrate (0.46 mg/kg), phosphate (18.24 mg/kg), TOC (31.4%), nickel (2.4 mg/kg), lead (1.83 mg/kg), and zinc (52.50 mg/kg). Baseline total heterotrophic bacteria (THB) and total hydrocarbon-utilizing bacteria (THUB) counts using standard microbiological methods were observed to be 3.11 × 10^8^ cfu/g and 2.43 × 10^8^ cfu/g, respectively.

### 3.2. Assessment and Evaluation of Degradation Potential

**Figure 4:**
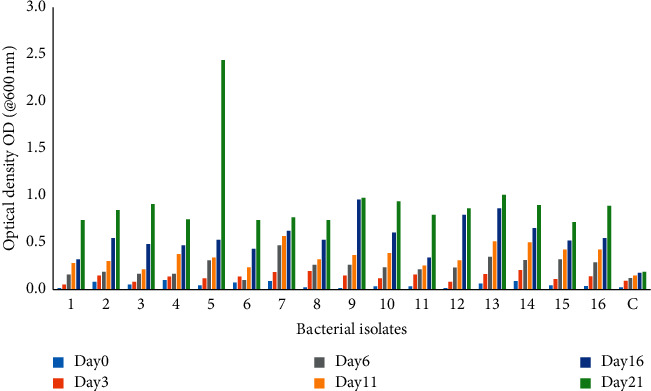
Biodegradation activity of bacterial isolates.

Degradation assay was conducted to determine the biodegradation potential of putative hydrocarbon-utilizing bacteria isolated from the crude oil-contaminated soil. The optical density readings were at variance across all bacterial isolates at different days. Isolates showed variations in the degradation rate of crude oil in a liquid broth medium, and this was scored based on visual observation of turbidity, emulsification of the crude oil, and optical density obtained with a spectrophototometer (600 nm wavelength), as presented in Figure 4. At the end of the experiment, a significant population of the bacteria isolates showed maximum degradation. These degrading capabilities discovered revealed that the microorganisms isolated from the polluted site were able to utilize crude oil as the source of energy and carbon. The cells were able to increase within the days of study, indicating utilization of oil for their growth and development and, hence, the concomitant increase in the concentration of the broth (turbidity).

Isolate HDB5 (*Bacillus cereus* strain Z-5) showed highest potential for crude oil degradation (2.450), while HDB 1 showed the least degradation efficiency (0.194). The control which contained just crude oil in the liquid broth medium without the test organism was 0.193 at day 21.

### 3.3. Bacterial Identification Based on Sanger Sequencing

Amplified PCR products (16S rRNA) were purified and sequenced. Electropherograms obtained from the sequence were called using chromas Lite version 2.01 software. Identification of bacterial 16S rRNA sequences was aligned with BLAST search facility of National Centre for Biotechnology information (NCBI) databases. The sequences aligned gave 99-100% similarity with those deposited in GenBank and as such considered close relatives and assigned identities. Sixteen (16) amplicons yielded good sequences whose identities have been assigned. The sequence result obtained is presented in Table 3. All isolates belonged to the domain bacteria. Bacteria isolates predominantly belonged to two (2) phyla, three (3) classes, and eleven (11) genera. The observed phyla, class, and genera and their percentage contributions to the whole are presented in subsequent pages. Nucleotide sequences obtained from this research have been deposited in GenBank database under accession numbers KX809608, KX809649, and KX809653 for all bacterial isolates identified except Cb27.  Table 4 illustrates a heatmap distribution of bacterial isolates across all microcosms in the different sampling days.

### 3.4. Functional Gene Diversity

The presence of hydrocarbon-degrading genes was assessed using PCR amplification with 5 specific primers targeting the PAH ring hydroxylating dioxygenase gene for Gram-positive bacteria (PAH-RHD_(GP)_), ring cleavage dioxygenase gene (catechol 2,3, dioxygenase), naphthalene dioxygenase (nahAC), long chain alkane degrading gene (alma), and midchain alkane monooxygenase (alkB). Biodegradation of n-alkanes and aromatic hydrocarbons efficiency were demonstrated with the presence of positive bands on gel visualized under a transilluminator after amplification of chromosomal and plasmid DNAs in the bacterial isolates. Specific detection of the five functional genes was achieved by combining PCR amplification with short oligonucleotides called primers. The primers target essentially the same regions on the genomic DNA and subsequently amplified with PCR to produce visible bands on gel electrophoresis.

The distribution of functional genes across all bacterial isolates generated is presented in a heatmap (Table 5). The chart also illustrates the number of genes present in each isolate and the location of the genes as regards to the genetic elements (plasmid or chromosome) harbouring the genes. AlkB and C23O were highly abundant and redundant in most of the isolates tested. Results revealed that an *Enterobacter hormaechei* strain PS53C and *Pseudomonas knackmussii* strain B13 harboured the highest number of genes across all screened isolates. Generally, Alk B and C230 were the most dominant genes present across all isolates.

## 4. Discussion

The physicochemical parameters tested in this study indicated long-term exposure of the sample to hydrocarbon contamination, and this was significantly evidenced in the low nitrate and phosphate values observed owing to the increase in the hydrocarbon content and a known effect of long-term hydrocarbon pollution. Likewise, the high concentrations of TPH and PAH values in the soil sample indicated substantial pollution of the test sample.

The putative oleophilic bacteria were isolated using the enrichment culture procedure [21, 24]. It was evident in this study that the utilization of the hydrocarbons by oleophilic bacteria resulted in visual gradual decrease in the crude oil layer in Bushnell Haas broth and a concomitant increase in optical density (absorbance) @ 600 nm wavelength. The growth dynamics of the organisms in the liquid medium as determined by O.D. showed that 60% of the isolates grew maximally in the medium with none exhibiting lag phases, and this could be attributed to genetic make-up of the individual bacteria and the constitutive expression of hydrocarbon catalyzing enzymes which may be lacking in the remaining 40%. This result is consistent with the findings of [21, 25, 26]. The poor growth rate and low O.D. reading observed in the 40% of the bacterial isolates could also be related to substrate specificity [27]. Some oleophilic bacteria are specific for degrading metabolites, by-products of crude oil degradation, or substrates not present in the crude oil composition, and this could have informed the low O.D. readings observed. This further proves that crude oil degradation is more effective and swift when microbes work in consortium (cometabolism) [28–30]. Patowary [30] stated that some members of the microbial community might have the ability to secrete important degradative enzymes and growth factors while others exhibit the potentiality of biosurfactant production leading to enhanced solubilization of hydrophobic hydrocarbons. The production of an extracellular biosurfactant is an underlying mechanism implemented by hydrocarbon degraders to mineralize petroleum hydrocarbons, and this was observed during the turbidometry assay by emulsification of crude petroleum in the broth culture inoculated with the test organisms. It has been repeatedly documented that different strains of *Bacillus* spp. found in different compartments of the environment produce biosurfactant/bioemuslifiers highly implicated in the degradation of a wide range of hydrocarbon compounds. Other isolates obtained in this study such as *Pseudomonas* spp., *Enterobacter* spp., *Burkholderia* spp., and *Acinetobacter* spp. have been previously documented as biosurfactant producers [31–33].

The presence of high enzymatic capacity allows microbial communities to degrade complex hydrocarbons (aliphatics and polyaromatics) and understanding the microbial functional diversity, and the factors influencing microbial functions is important for bioremediation studies [34]. To underpin the roles of the different bacterial genera isolated from the polluted soil sample and to estimate the genetic diversity contributing to the versatility in the bacterial transformation of hydrocarbon pollutants, bacterial community functional structure was analyzed using five specific genes, namely, alkB, Alma, C230, PAH-RHD_(GP)_, and nahAC. Hydrocarbon-utilizing bacteria isolated from the sample showed that about 50% of the organisms possessed alkane and catechol 2,3, dioxygenase genes harboured independently in different locations especially the plasmid, while some bacteria possessed specific genes in both locations. In functional genomics, the knowledge of the location of the genes or the genetic element harbouring genes is very important because it serves as a contributing factor to rapid microbial community evolution or adaptation after a spill or environmental perturbation. Both chromosomes and plasmids are genetic elements that harbour functional genes; however, while the former acts as insertion elements, the latter acts as the mobile genetic element facilitating horizontal gene transfer [18–20, 35]. Shintani and Nojiri [36] stated that mobile genetic elements (MGEs) are important “vehicles” of diverse genes in the microbial genetic pool. Exchange of MGEs in the microbial community confers new characters to their hosts and encourages swift adaptation to various environments. This explains how over 90% of the oleophilic bacteria isolated from the aged spill site harboured diverse functional genes, especially alkB and C23O. This result corresponds with the findings documented by [37–39].

*Pseudomonas knackmussii* B13 was the only bacteria that harboured the nahAC gene in the plasmid, while PAH-RHD_(GP)_ was present in an unidentified bacterium. The PAH-RHD_(GP)_ are ring hydroxylating genes present in Gram-positive bacteria, and in this study, most of the bacteria isolated were Gram-negative, and this contributed to the presence of one bacterium harbouring the gene. However, a more improved primer system has been developed that targets both PAH-RHD*α* genes for both Gram-positive and Gram-negative [40]. Generally, 90% of the total bacteria isolated from each microcosm possessed at least a gene and some harboured two or three genes each, while an appreciable number had specific genes located in both the plasmid and chromosome. Most of the genes were located in the plasmid, and this is consistent with the report of Mirdamadia et al. [41] and Shahi et al. [42] which states that the catabolic pathways encoding degradation routes of different aromatic and aliphatic hydrocarbons are frequently located on plasmids. However, oleophilic bacteria isolate *Acinetobacter baumannii* strain 578CR did not harbour any of the five functional genes tested, although turbidometry screening revealed otherwise. *Acinetiobacter* is known to be among microbial communities involved in different ecosysytem functions. Several strains of this genus have been attracting growing attention, particularly in bioremediation, especially *A. baumannii* and *A*. *calcoaceticus* [43].

The functional diversity reportedly revealed the presence of catabolic genes that are responsible for aliphatic and aromatic hydrocarbon degradation. In this study, five (5) enzymes coding degradative genes including monooxygenase and dioxygenase responsible for the degradation of petroleum hydrocarbons were detected at varied proportions across all isolates. This implies that the inhabiting bacterial population are not only passive or randomly present in the sample but are also actually influenced by the influx of crude oil into the system via pollution [44]. For alkB, the chemical and physicochemical composition of the soils, such as pH, TPH, organic matter, and/or plant litter, may have influenced the diversity and richness of alkB phylotypes, as suggested by other studies [45–47]. Species of *Pseudomonas aeruginosa* strain A-1, *Staphylococcus xylosus* strain AV1, *Bacillus thuringiensis* strain VKK-BB-2, *Pseudomonas aeruginosa* strain VV163, *Burkholderia cepacia* strain KHD 08, *Pseudomonas knackmussii* strain B13, *Burkholderia ambifaria* strain R1, *Enterobacter hormaechei* strain PS53C, and *Acinetobacter nosocomialis* strain: GTC 03313 harboured the alkB gene. M'rassi et al. [48] isolated and characterized different bacteria strains for bioremediation of environments contaminated with n-alkanes. Bacterial isolates obtained were 150 and mainly affiliated to the Gammaproteobacteria class while the alkB gene was detected in about 80% of the bacterial strains. Alkanes constitute a higher fraction of the TPH concentration (55%), and this justifies the global interest in the distribution of alkB in the environment. However, Jurelevicius et al. [47] reported that the diversity of the alkB gene in environmental samples is far from being well studied. The report highlighted that the use of a single pair of primers for PCR quantification of the alkB gene in soil environments limits the range of detection of the genes present. This could be attributed to variations in alkB nucleotide sequences within a bacterial community, and as such, failure to consider the variations will result in a low representation of the diversity and richness of the alkane degrading gene. Shahi et al. [44] evaluated the microbial population and functional genes during the bioremediation of petroleum-contaminated soil as an effective monitoring approach, and the report captured that Gram-negative bacteria and alkB are critical to successful bioremediation. Also, the report stated that failure to maintain the stability of oleophilic bacteria and functional genes will reduce the extent to which alkanes and PAHs are degraded. Additionally, results showed that Alma genes were harboured in five bacterial isolates across and the genes were all located in the plasmid. This is indicative that these organisms have the potential to mineralize long chain alkanes although research has revealed that the Alma gene is more likely to be found in marine hydrocarbon degrading bacterial than terrestrial [49].

The catechol dioxygenase class of bacterial iron-containing enzymes is an example of enzymes implicated in the aerobic mineralization of aerobic aromatic hydrocarbons. This enzyme is involved in aromatic ring cleavage and is responsible for the wide variety of microorganisms that are capable of degrading aromatic compounds [50]. This study witnessed a rapid response to PAHs stress after crude oil contamination. The catabolic gene survey confirmed the enrichment of indigenous community as a result of the influx of high concentration of PAH into the soil during crude oil pollution. Thomas et al. [51] detected C23O in isolates affiliated with three closely related taxonomic subgroups within the *Pseudomonas* group. He et al. [52] characterized the diversity and distribution pattern of C23O in surface sediments of the Bohai sea. The results showed that sediments of the Bohai sea were dominated by genes related to C23O. Kasuga et al., Olukunle et al., and Baruah et al. [53–55] detected the presence of the C23O gene in bacteria isolated from different environments polluted with petroleum hydrocarbons. The presence of these degradative genes in the various hydrocarbon-utilizing bacteria isolated from the polluted site indicates the possible application of these microbes in bioremediation and ecorestoration of crude oil-polluted sites.

## 5. Conclusions

In this study, the biodegradability potential of indigenous hydrocarbon-degrading bacteria isolated from an aged spill site was detected by turbidometry assay. *Bacillus cereus* strain Z-5 showed the highest potential amongst other isolates tested. The functional gene diversity of the isolates revealed diverse catabolic genes (aliphatic and aromatic) residing in the plasmids, chromosomes, and, in some cases, both plasmid and chromosomes of the organisms. However, the C230 gene was resident in >50% of the microbial population tested. This report reveals that there is no direct relationship between the biodegradability potential of microbes in a liquid mineral salt broth medium and functional gene diversity. For example, *Bacillus cereus* strain Z-5 which had the highest optical density value in turbidometry screening only harboured one gene (Alma) amongst the five genes used in this study although other possible genes not accounted for could be the reason for such degrading potential displayed. The results reveals that the crude oil polluted site in komkom community, Rivers State, Nigeria, is a hub for hydrocarbon degrading organisms, and since most the genes that codes for this function were shown to be resident in the plasmid, there is a strong likelihood of horizontal gene transfer occurring at the site and a possible increase in the population of hydrocarbon degraders over time. This information could be harnessed as baseline for a more improved study and possible remediation of the site.

## Figures and Tables

**Table 1:** Polycyclic aromatic hydrocarbon components of soil.

PAH component	Conc.
Benzo(b)fluoranthene	56.94305
Acenaphthylene	45.06849
Acenaphthene	58.76505
Benzo(k)fluoranthene	78.93263
Fluorene	197.61813
Fluoranthene	–
Benz(a)anthracene	115.83669
Indeno(1, 2, 3-d)pyrene	67.86253
Dibenz(a,h)anthracene	26.68901
Pyrene	100.62592
Phenanthrene	78.84827
Chrysene	20.32951
Naphthalene	17.76326
Benzo(a)pyrene	45.06849
Benzo(g, h, i)perylene	25.16782
Anthracene	13.00487
**Total**	**989.11889**

**Table 2:** Total petroleum hydrocarbon components of the polluted soil.

TPH	Conc. (mg/kg)
c8	—
c9	—
c10	1116.36824
c11	423.27828
c12	155.13926
c13	509.20511
c14	—
c15	—
c16	—
c17	—
c18	—
c19	131.58471
c20	110.71325
c21	0.00000
c22	827.41236
c23	0.00000
c24	2006.25633
c25	—
c26	105.51325
c27	0.00000
c28	390.47122
c29	1294.12988
c30	1132.60042
c31	249.33617
c32	—
c33	—
c34	115.54043
Total	**8695.7723**

**Table 3:** Baseline properties/characteristics of the study site.

Parameters	Method	DPR intervention limit	Concentration/quantity
pH	APHA 4500H^+^B	NA	5.97
E. conductivity (*μ*s/cm)	Cond. Meter	NA	60
Moisture content (%)	BS 1377-2:1990 clause 1	NA	2.40
Nitrate (NO_3_) (mg/kg)	APHA 4500- NO^3−^	NA	0.46
Phosphate (P_2_O_5_) (mg/kg)	APHA 4500-P	NA	18.24
Total organic carbon (%)	BS 1377-2:1990 clause 3	NA	31.4
Nickel (mg/kg)	ASTM D 1886	10	2.420
Lead (mg/kg)	ASTM D 3559	380	1.283
Zinc (mg/kg)	ASTM D 1691	210	52.50
TPH (mg/kg)	EPA 8015	5000	8695.77231
PAH (mg/kg)	EPA 8260	40	989.11889
THB	Spread plate	NA	3.11 × 10^8^
HUB	Spread plate	NA	2.43 × 10^8^

Method source: the American Public Health Association (APHA) 20^th^ Edition 2008, American Society for for Testing and Materials (ASTM), EPA: Environmental Protection Agency. Keys: Cond. Meter: conductivity meter, NA: not available, BS: British Standard, Cfu/g: colony forming unit per gram.

**Table 4:** The 16S rRNA identification of bacterial isolates.

Isolate code	Accession number	Closest relative accession no.(bases compared)	Similarity (%)	Phylogenetic	Affiliation
				Phylum	Bacterial identity
Cb1	KX809626	KT021522.1	100	Proteobacteria	*Enterobacter hormaechei* strain SDI-42
Cb2	KX809627	KX426044.1	100	Proteobacteria	*Pseudomonas aeruginosa* strain A-1
Cb3	KX809628	KU726513.1	99	Firmicute	*Staphylococcus xylosus* strain AV1
Cb4	KX809629	KX390638.1	100	Firmicute	*Staphylococcus hominis* strain H69
Cb5	KX809630	KR697783.1	100	Firmicute	*Bacillus cereus* strain Z-5
Cb6	KX809631	KT714045.1	100	Firmicute	*Bacillus thuringiensis* strain VKK-BB-2
Cb7	KX809634	CP014647.1	99	Proteobacteria	*Klebsiella pneumoniae* strain KPNIH36
Cb8	KX809617	KR349544.1	99	Proteobacteria	*Pseudomonas aeruginosa* strain VV163
Cb9	KX809636	KC878000.1	100	Proteobacteria	*Burkholderia ambifaria strain R1*
Cb10	KX809638	NR_121733.1	100	Proteobacteria	*Pseudomonas knackmussii* strain B13
Cb11	KX809641	KX289657.1	100	Proteobacteria	*Pseudomonas stutzeri* strain SMG-8_NRB-DRDO MP
Cb12	KX809642	KX214111.1	100	Proteobacteria	*Acinetobacter baumannii* strain 578CR
Cb13	KX809644	KT717633.1	100	Proteobacteria	*Burkholderia cepacia* strain KHD 08
Cb14	KX809613	JX294308.1	99	Proteobacteria	*Enterobacter hormaechei* strain PS53C
Cb15	N/A	N/A	N/A	N/A	N/A
Cb16	KX809623	LC014122.1	100	Proteobacteria	*Acinetobacter nosocomialis* strain: GTC 03313

**Table 5:** Heatmap of functional gene diversity of bacterial isolates located in the plasmid and chromosome.

S/no.	Isolates	Alk B	Alma	PAH-RHD_(GP)_	C230	Nah AC
Cb1	*Enterobacter hormaechei* strain SDI-42/		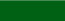			
Cb2	*Pseudomonas aeruginosa* strain A-1	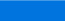			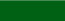	
Cb3	*Staphylococcus xylosus* strain AV1	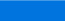	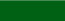			
Cb4	*Staphylococcus hominis* strain H69/		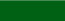			
Cb5	*Bacillus cereus* strain Z-5		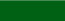			
Cb6	*Bacillus thuringiensis* strain VKK-BB-2	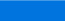			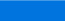	
Cb7	*Klebsiella pneumoniae* strain KPNIH36			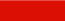		
Cb8	*Pseudomonas aeruginosa* strain VV163	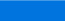				
Cb9	*Burkholderia cepacia* strain KHD 08	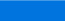				
Cb10	*Pseudomonas knackmussii* strain B13	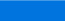	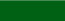		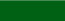	
Cb11	*Pseudomonas stutzeri* strain SMG-8_NRB-DRDO MP				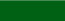	
Cb12	*Acinetobacter baumannii* strain 578CR					
Cb13	*Burkholderia ambifaria* strain R1	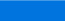			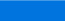	
Cb14	*Enterobacter hormaechei* strain PS53C	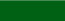			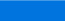	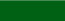
Cb15	Nil	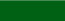			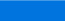	
Cb16	*Acinetobacter nosocomialis* strain: GTC 03313	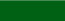			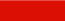	
*Key*						
Plasmid						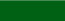
Chromosome						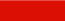
Plasmid + chromosome						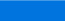

## Data Availability

The data (tables and figures) used to support the findings of this study are included within the article.

## Supplementary Materials

Supplementary Materials PCR conditions for functional genes in plasmid and chromosomal DNA: five functional degradative genes targeting alk B (short and middle chain alkanes, C_8_–C_16_), Alma (long chain alkane, C_16_–C_36_), catechol 2, 3 dioxygenase (aromatic hydrocarbons), PAH-RHD_(GP)_ (aromatic hydrocarbons), and nah AC (naphthalene-degrading gene) were used to detect these genes in the putative hydrocarbon-utilizing bacteria to determine their catabolic potentials and capabilities. Also, all isolates were analyzed for plasmid and chromosomal DNA to determine the location of the genes. The process involved PCR with the specific genes targeting specific sites on the bacterial genomic and plasmid DNA molecules responsible for the breakdown of different crude oil constituents. The PCR programme for all functional gene amplifications is shown in Tables 1–4.
