# Supplementary material for: Functional Gene Diversity of Selected Indigenous Hydrocarbon-Degrading Bacteria in Aged Crude Oil
Source: Int J Microbiol. 2020 Jul 30;2020:2141209. doi: 10.1155/2020/2141209 (PMC7414327; doi:10.1155/2020/2141209)
Supplement: Supplementary Materials — PCR conditions for functional genes in plasmid and chromosomal DNA: five functional degradative genes targeting alk B (short and middle chain alkanes, C8–C16), Alma (long chain alkane, C16–C36), catechol 2, 3 dioxygenase (aromatic hydrocarbons), PAH-RHD(GP) (aromatic hydrocarbons), and nah AC (naphthalene-degrading gene) were used to detect these genes in the putative hydrocarbon-utilizing bacteria to determine their catabolic potentials and capabilities. Also, all isolates were analyzed for plasmid and chromosomal DNA to determine the location of the genes. The process involved PCR with the specific genes targeting specific sites on the bacterial genomic and plasmid DNA molecules responsible for the breakdown of different crude oil constituents. The PCR programme for all functional gene amplifications is shown in Tables 1–4. [file 2141209.f1.docx]

**Supplementary Tables**

**Table 1 PCR condition for amplification of catechol 2, 3 – dioxygenase gene in plasmid and chromosome**

| **Process** | **Temperature (^o^C)** | **Time** |
| --- | --- | --- |
| Initial denaturation | 95 | 3 min |
| Denaturation | 95 | 30 s |
| Annealing | 52 | 40 s |
| Extension | 72 | 45 s |
| Final extension | 72 | 2 min |
| Cooling and holding time | 4 | **∞** |

**Table 2 PCR condition for amplification PAH – RHD_(GP)_ and nah AC gene in plasmid and chromosome**

| **Process** | **Temperature (^o^C)** | **Time** |
| --- | --- | --- |
| Initial denaturation | 95 | 5 mins |
| Denaturation | 94 | 40 secs |
| Annealing | 55 | 40 secs |
| Extension | 72 | 1 min |
| Final extension | 72 | 7 mins |
| Cooling and holding time | 4 | ∞ |

**Table 3 PCR condition for amplification Alma gene in plasmid and chromosome**

| **Process** | **Temperature** | **Time** |
| --- | --- | --- |
| Initial denaturation | 94 | 5 min |
| Denaturation | 94 | 30 secs |
| Annealing | 50 | 30 secs |
| Extension | 72 | 60 secs |
| Final extension | 72 | 10 mins |
| Cooling and holding time | 4 | ∞ |

**Table 4 PCR condition for amplification of alk B gene in plasmid and chromosome**

| **Process** | **Temperature (^o^C)** | **Time** |
| --- | --- | --- |
| Initial denaturation | 95 | 7 mins |
| Denaturation | 94 | 30 secs |
| Annealing | 53 | 1 min |
| Extension | 72 | 1 min |
| Final extension | 72 | 7 mins |
| Cooling and holding time | 4 | ∞ |
